# Temperature has an overriding role compared to photoperiod in regulating the seasonal timing of winter moth egg hatching

**DOI:** 10.1007/s00442-024-05535-w

**Published:** 2024-03-23

**Authors:** Natalie E. van Dis, Lucia Salis, Marcel E. Visser

**Affiliations:** 1 Department of Animal Ecology, Netherlands Institute of Ecology (NIOO-KNAW), P.O. Box 50, 6700 AB Wageningen, The Netherlands; 2 Groningen Institute for Evolutionary Life Sciences, University of Groningen, 9747 AG Groningen, The Netherlands; 3 Helsinki Institute of Life Science, University of Helsinki, P.O. Box 4, 00014 Helsinki, Finland

**Keywords:** Climate change, Phenological mismatch, Insect dormancy, Diapause, *Operophtera brumata*

## Abstract

To accurately predict species’ phenology under climate change, we need to gain a detailed mechanistic understanding of how different environmental cues interact to produce the seasonal timing response. In the winter moth (*Operophtera brumata*), seasonal timing of egg hatching is strongly affected by ambient temperature and has been under strong climate change-induced selection over the past 25 years. However, it is unclear whether photoperiod received at the egg stage also influences timing of egg hatching. Here, we investigated the relative contribution of photoperiod and temperature in regulating winter moth egg development using two split-brood experiments. We experimentally shifted the photoperiod eggs received by 2–4 weeks compared to the actual calendar date and measured the timing of egg hatching, both at a constant temperature and in combination with two naturally changing temperature treatments – mimicking a cold and a warm year. We found an eight-fold larger effect of temperature compared to photoperiod on egg development time. Moreover, the very small photoperiod effects we found were outweighed by both between- and within-clutch variation in egg development time. Thus, we conclude that photoperiod received at the egg stage does likely not play a substantial role in regulating the seasonal timing of egg hatching in the winter moth. These insights into the regulatory mechanism of seasonal timing could have important implications for predicting insect climate change adaptation, as we might expect different targets of selection depending on the relative contribution of different environmental cues.

**Supplementary Information:**

The online version contains supplementary material available at 10.1007/s00442-024-05535-w.

## Introduction

The seasonal timing of a wide range of species is shifting in response to climate change (Parmesan and Yohe 2003; Root et al. 2003; Thackeray et al. 2010), largely in response to increasing temperatures (Cohen et al. 2018). In many cases, interacting species are shifting their phenology at different rates, with the resulting phenological mismatches between consumer and resource leading to natural selection on phenology and possibly negative consequences for population viability (Kharouba et al. 2018; Visser and Gienapp 2019). The relative importance of different environmental cues, such as temperature and photoperiod, to time key life history events is generally thought to play a role in determining a species’ seasonal timing shift (Chmura et al. 2019; Renner and Zohner 2018). However, to accurately predict species’ responses to climate change, we need to gain a detailed mechanistic understanding of how different environmental cues interact to produce the seasonal timing response (Chmura et al. 2019; McNamara et al. 2011).

In many species, the seasonal timing of life history events is under photoperiodic control, with additional environmental cues such as temperature used to fine-tune the response (Bradshaw and Holzapfel 2007). In insects, photoperiod similarly plays a major role in determining the timing of development, particularly in regulating dormancy responses (Danks 1987; Denlinger 2002). While photoperiod is primarily involved in the induction of diapause in insects (Denlinger 2002), it can similarly act as a cue for diapause maintenance (Tauber and Tauber 1976) and diapause termination (Brunnarius and Dumortier 1984; Koštál et al. 2017). For example, in the European corn borer (*Ostrinia nubilalis*) and Asian corn borer (*Ostrinia furnacalis*), larvae that have entered diapause remain sensitive to photoperiod throughout autumn and early winter, with day length in combination with temperature regulating the duration of diapause (McLeod and Beck 1963; Yang et al. 2014). Interestingly, due to this interaction between photoperiod and temperature to regulate seasonal timing, adaptation to climate change could also involve changes in a species’ sensitivity to photoperiod rather than just temperature (Bradshaw and Holzapfel 2006). For example, the pitcher plant mosquito (*Wyeomyia smithii*) and the speckled wood butterfly (*Pararge aegeria*) have genetically adapted their response to photoperiod to exploit the longer growing season under climate change (Bradshaw and Holzapfel 2001; Nielsen et al. 2023).

In the winter moth (*Operophtera brumata*), climate change adaptation seems to have primarily involved genetic changes to the temperature sensitivity of egg development rate (van Asch et al. 2013). Winter moths are distributed across Europe (Spain to Northern Scandinavia; (Tenow et al. 2013) and are univoltine, with adults emerging and laying eggs in early winter (November/December). Eggs then go through a long dormancy period of several months, as they need to hatch in early spring to feed for 4–6 weeks on young leaves until pupation (Salis et al. 2017). In the Netherlands, warmer winter and spring temperatures experienced during the egg dormancy period advanced the seasonal timing of egg hatching to such an extent that winter moth caterpillars emerged more than 10 days before their food source, young oak leaves, became available (van Asch et al. 2013). Previous work has shown that the temperature response of winter moth egg development rate is genetically determined and has shifted over time in response to this strong selection pressure, leading to a better phenological match between the timing of egg hatching and the timing of oak budburst (van Asch et al. 2007, 2013).

While in many species egg diapause involves a period of developmental arrest which can only be broken by low temperatures – e.g. in the streak moth, *Chesias legatella* (Wall 1973), and the silk moth, *Bombyx mori* (Niimi et al. 1993) – in the winter moth, the completion of egg development does not require low temperatures. Instead, egg development is continuous with embryos remaining temperature sensitive throughout development (van Dis et al. 2021) – similar to, for example, the pea aphid, *Acyrthosiphon pisum* (Shingleton et al. 2003). Since winter moth eggs are exposed to photoperiod during development in natural conditions (Varley et al. 1973) and photoperiod can be involved in the induction, maintenance, and termination of insect diapause (Denlinger 2002; Tauber and Tauber 1976), winter moth eggs might similarly be sensitive to photoperiod during development. However, it remains unknown whether photoperiod also plays a role in regulating the timing of winter moth egg dormancy, and if so, how it interacts with temperature.

Here, we investigated whether photoperiod received at the egg stage influences the seasonal timing of egg development in the winter moth, both as a cue on its own and in interaction with temperature. In two split-brood experiments, we determined egg development time after giving eggs either an early or late season photoperiod treatment, with naturally changing day lengths shifted 2–4 weeks earlier or later compared to the actual calendar date. Temperature was kept constant in the first experiment, while the second experiment also incorporated two naturally changing temperature treatments – mimicking a cold and a warm year – to investigate the relative contribution of temperature and photoperiod. If winter moth eggs are sensitive to photoperiod during development, we expected eggs that received a late season photoperiod to hatch earlier than eggs that received an early season photoperiod. Elucidating which environmental cues regulate the seasonal timing of egg hatching in the winter moth is essential for understanding its response to climate change.

## Methods

### Photoperiod experiment

**Fig. 1:**
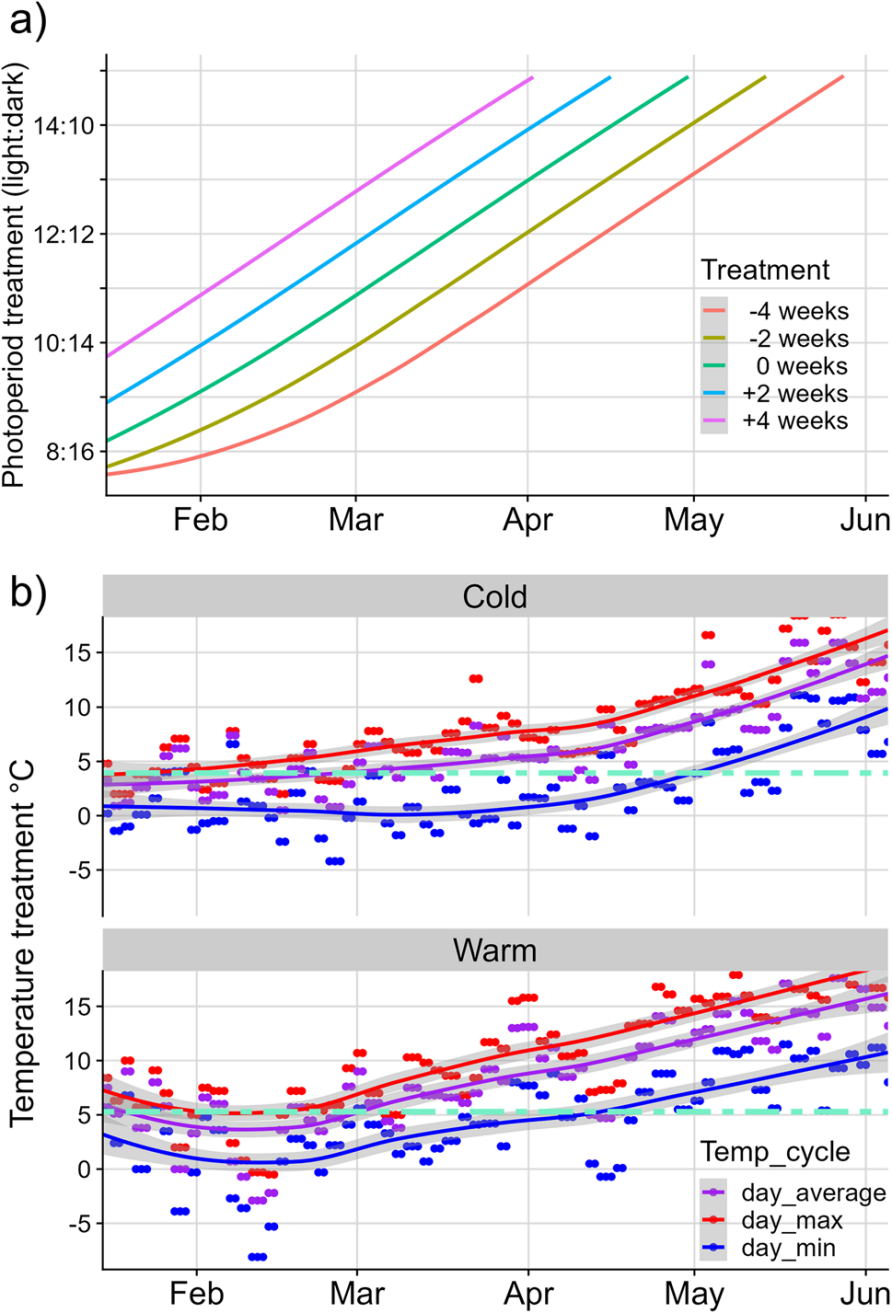
Experimental photoperiod and temperature treatments. In two split-brood experiments, eggs received either an early or late season photoperiod treatment, with naturally changing day lengths shifted 2–4 weeks earlier or later compared to the actual calendar date (panel a, showing day lengths in hours for each treatment over the course of the experiment; [0 weeks] = control treatment). Temperature was kept constant in the first experiment, while the second experiment also incorporated two naturally changing temperature treatments –mimicking a cold and a warm year (panel b). Temperatures were changed daily with each day using a three-phase temperature cycle: this cycle consisted of 6 h at the daily minimum temperature (blue), 12 h at the mean of the daily maximum and the daily average (red), and 6 h at the daily average for each year (purple). The turquoise line gives the average observed temperature for each treatment (Table S3). Refer to the main text for more details on the experiments. Photoperiod and temperature treatments used in the experiments can be found on Dryad (van Dis et al. 2024)

In the first of two split-brood experiments, we aimed to determine the effect of photoperiod on the seasonal timing of winter moth egg hatching at a constant temperature. Eggs were collected in 2013 from 31 wild winter moth females caught in a forest near Oosterhout, the Netherlands (Catch dates: November 25–29, (van Asch et al. 2013). The 31 clutches of eggs (ranging from 221 to 341 eggs) were kept in the dark in an outside field shed until the start of the experiment (January 15, 2014). For the experiment, we manipulated the photoperiod that the eggs received, using photoperiod treatments from a previous study (Salis et al. 2017). Each photoperiod treatment followed the naturally changing day length in the field, but the day length was shifted either 2 or 4 weeks compared to the actual calendar date. In total, we had five photoperiod treatments: (1) control [0 weeks shift], (2) very early season photoperiod [−4 weeks], (3) early season photoperiod [−2 weeks], (4) late season photoperiod [+ 2 weeks], and (5) very late season photoperiod [+ 4 weeks], with a maximal day length difference of 3.8 h between the [−4 weeks] and [+ 4 weeks] treatments in mid-March (photoperiod treatments over the season visualized in Fig. 1). Each female’s clutch was split into sub-clutches of at least 10 eggs (by cutting the substrate paper eggs were laid on), and divided over the five photoperiod treatments, assigning three sub-clutches per female to each treatment (i.e. three replicates, 3 sub-clutches × 5 treatments × 31 females = 465 sub-clutches in total). All sub-clutches were kept in the same climate room at a constant 10 °C (temperature monitored with loggers, Thermochron iButton), but each replicate per treatment was housed in an individual, ventilated box (i.e. 15 boxes, 30 × 30x30cm; Fig. S1) equipped with a light bulb (Goobay LED 2W/6200 K, white, 1095–1132 lx as measured at the bottom of the boxes). Eggs thus experienced the same temperature but were exposed to different photoperiod treatments. Egg hatching was checked three times a week, from which we determined the date at which 50% of each sub-clutch had hatched (D50). D50 was not determined for sub-clutches in which less than 10 eggs hatched (3 sub-clutches excluded, each from different females and treatments [−4 weeks, + 2 weeks, 0 weeks]).

### Photoperiod–temperature experiment

In a second split-brood experiment, we determined the relative contribution of photoperiod and temperature in regulating the seasonal timing of winter moth egg hatching. The experiment consisted of two photoperiod treatments, early season photoperiod [−2 weeks] and late season photoperiod [+ 2 weeks], with a maximal day length difference of 2 h in mid-March, and two temperature treatments, mimicking a cold year [1973] and a warm year [1999] with an average temperature difference of 1.36 °C (Fig. 1), in a full factorial design (two photoperiod x two temperature = four treatments in total). Temperature treatments were replicated from a previous study (Visser and Holleman 2001), where the temperature that the eggs received was changed daily with each day using a three-phase temperature cycle; this cycle consisted of 6 h at the daily minimum temperature, 12 h at the mean of the daily maximum and the daily average, and 6 h at the daily average for each year (see Fig. 1; temperatures used can be found on Dryad (van Dis et al. 2024). Eggs were collected in 2000 from 20 wild winter moth females also caught in the Oosterhout forest (see above; Catch dates: November 20–27). At the start of the experiment (December 12, 2000), the 20 clutches of eggs (ranging from 59 to 163 eggs) were divided into sub-clutches over four climate cabinets (4 treatments × 20 females = 80 sub-clutches; one cabinet per treatment; SANYO Incubator, Model: MIR-553), equipped with a light source (Philips TL mini 8W/33 T, 640 white, 400–500 lx) and loggers to monitor temperature (see above). Egg hatching was checked twice a week to determine D50 for each sub-clutch in the following spring, excluding one sub-clutch from the warm-[+ 2 weeks] treatment in which less than 10 eggs hatched (see above).

### Statistical analysis

We used linear mixed models to test for the effects of photoperiod and temperature on egg development duration in R v.4.31 (R Core Team 2023) with packages lme4 v.1.1–34 (Bates et al. 2015) and lmerTest v.3.1–3 (Kuznetsova et al. 2017), at a significance level of *α* = 0.05. Egg development duration was calculated per sub-clutch as the duration in days from female catch date (i.e. proxy for the day the clutch was laid) to D50 hatching date. For the photoperiod experiment, photoperiod treatment was included as a fixed effect, and FemaleID as random effect including a random slope for treatment. Treatment random slopes were only retained in the model if significant (*P* < 0.05, method: likelihood ratio test [LRT] with lmerTest::ranova). For the photoperiod–temperature experiment, we included photoperiod and temperature treatment as fixed effects, as well as the interaction between temperature and photoperiod, and FemaleID as random effect, but not fitting a random slope (as there were less than 4 treatments with replicates). Checking the model residuals revealed one major outlier in the photoperiod–temperature experiment (> 3SD above the mean, Fig.S2), resulting from one female’s sub-clutch having a very different egg development time compared to her other sub-clutches. We excluded this outlier from the analysis, which slightly changed the estimates, but not the direction or significance of the results (all data and code deposited, (van Dis et al. 2024). For both experiments, we performed post hoc tests for significant fixed effects using R package emmeans v.1.8.8 (Lenth 2023), comparing photoperiod and temperature treatments and their interaction. For the photoperiod experiment, we also performed an ordered heterogeneity (OH) test, assessing whether the mean hatching dates per treatment followed the expected order of late to early hatching: [−4 weeks] > [ −2 weeks] > [0 weeks] > [+ 2 weeks] > [+ 4 weeks].

## Results

### Photoperiod experiment

**Fig. 2:**
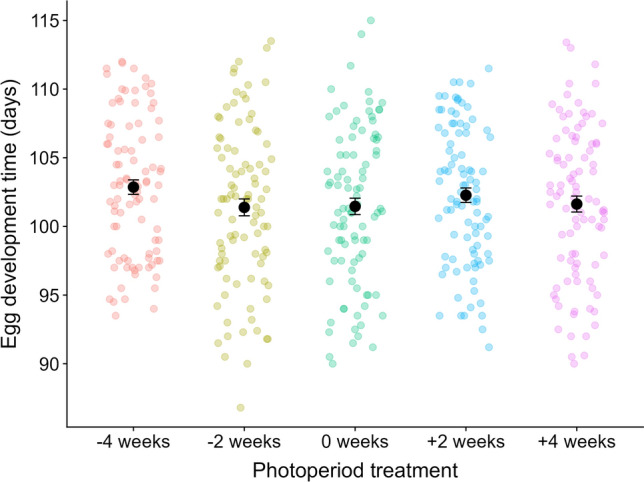
Minor effect of photoperiod on egg development time. Mean egg development times in days ± Standard Error (SE) are shown for each photoperiod treatment. Observed egg development times for each sub-clutch are plotted in the background, coloured by photoperiod treatment. Effect sizes can be found in the main text and the Supplements (Table S2)

We found large variation in egg development time (similar to previous studies, e.g. (van Dis et al. 2021), with a small mean effect of photoperiod (Fig. 2, *P* < 0.001, photoperiod effect estimates = −0.88 to 1.48 days, Table S1–2). Post hoc tests showed that only the [−4 weeks] treatment (i.e. very early season photoperiod) differed from the other treatments (*P* < 0.05; except for [+ 2 weeks] treatment *P* = 0.59, Table S2). Although the effect was in the expected direction, this four-week shift in photoperiod – amounting to a maximum difference in day length of 3.8 h in mid-March between the [−4 weeks] and [+ 4 weeks] treatments (Fig. 1) – led to eggs on average hatching only 1.4 days later than the other treatments, and could alternatively be explained by small unintended temperature differences between photoperiod treatments (all treatments constant 10 °C ± 0.1–0.3, Table S3). Moreover, the small effect size of photoperiod was outweighed by the variation in egg development time between clutches (significant random intercept effect: LRT = 507.03, *P* < 0.001; but not random slope: LRT = 4.85, *P* = 0.99), with mean egg development time per female ranging from 94 to 110 days (16 days max. difference) and no clear photoperiod effect visible per clutch (Fig.S3). Mean hatching dates of the photoperiod treatments also did not follow the expected order effect of late to early hatching ([−4 weeks] > [ −2 weeks] > [0 weeks] > [+ 2 weeks] > [+ 4 weeks]; OH test = −0.20, *P* > 0.1).

### Photoperiod–temperature experiment

**Fig. 3:**
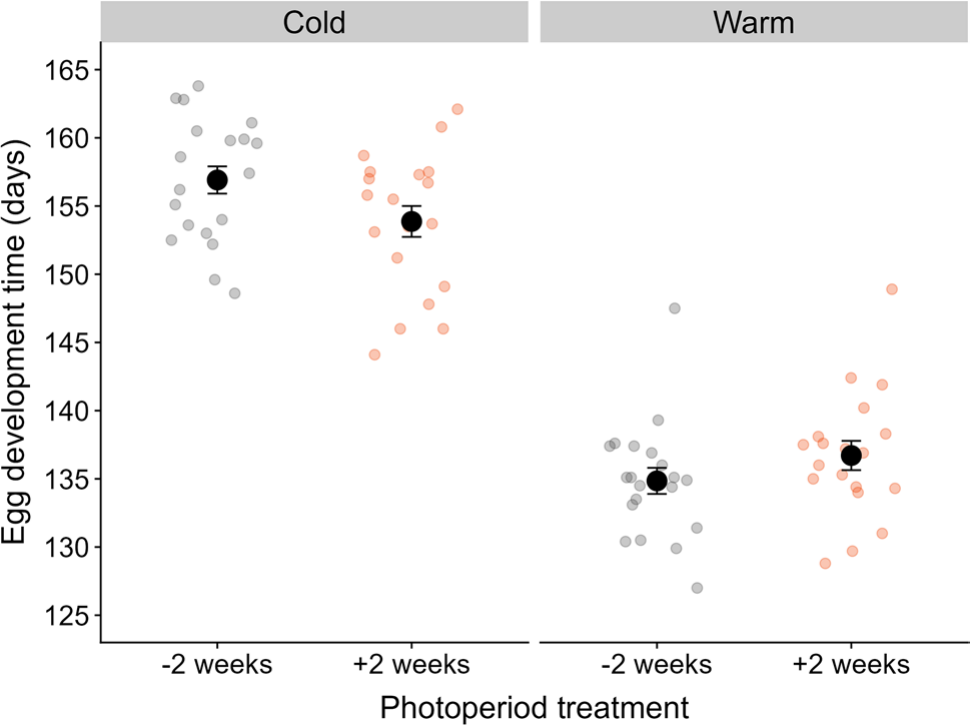
Eight-fold larger effect of temperature compared to photoperiod on egg development time. Mean egg development times ± SE are plotted for each photoperiod and temperature treatment combination, with observed egg development times per sub-clutch plotted in the background and coloured by photoperiod treatment. See for effect sizes the main text and the Supplements (Table S4)

Our analysis indicated an interaction effect of photoperiod and temperature on egg development time (Fig. 3, *P* < 0.001, Table S1). Comparing the photoperiod treatments within temperature treatments showed a small effect of photoperiod on egg development time, but only in the cold treatment with eggs in the [− 2 weeks] photoperiod treatment hatching slightly later (cold treatment: estimate = 2.49 days, *P* < 0.001; warm treatment: estimate = −1.57, *P* = 0.053, Table S4). This effect is in the expected direction similar to what we observed in the photoperiod experiment (Fig. 2), but also in this experiment there were small unintended mean temperature differences between treatments that could alternatively explain the observed difference in egg development time (Table S4): the [− 2 weeks]-cold treatment (mean [12Dec to median hatching date] = 3.89 °C) was on average slightly colder than the [+ 2 weeks]-cold treatment (mean = 3.98 °C); while the [−2 weeks]-warm treatment was on average slightly warmer (mean = 5.48 °C) than the [+ 2 weeks]-warm treatment (mean = 5.10 °C).

In contrast to the small photoperiod effect, we found an eight-fold larger effect of temperature on egg development time – for an average temperature difference of 1.36 °C between the warm and the cold treatments – with eggs on average hatching 19.92 days earlier in the warm compared to the cold treatment (*P* < 0.001, Tables S1 and S4). Also in this experiment, we observed large variation in egg development time between clutches (significant random intercept effect: LRT = 72.69, *P* < 0.001), with mean egg development time per female ranging from 137 to 155 days (18 days max. difference), but the temperature effect was clearly visible for each clutch (Fig.S4).

## Discussion

To accurately predict species’ responses to climate change, we need to understand how different environmental cues interact to produce the seasonal timing response (Chmura et al. 2019; McNamara et al. 2011). In the winter moth, seasonal timing of egg hatching is strongly influenced by ambient temperature resulting in severe climate change-induced selection over the past 25 years (van Asch et al. 2013). However, it was unclear whether photoperiod also influences egg development duration. Here, we investigated the relative contribution of photoperiod and temperature in regulating winter moth egg development using two split-brood experiments. We found an eight-fold larger effect of temperature compared to photoperiod on egg development time.

Photoperiod as a cue did not affect egg development time as expected. While the photoperiod treatments we used can induce strong shifts (10–20 days) in the seasonal timing of winter moth adult emergence when applied during the caterpillar stage (Salis et al. 2017), in the experiments performed here, we only found a photoperiod effect on egg development timing in some of the administered treatments. Egg development time was affected only in the (very) early season photoperiod treatment (i.e. photoperiod delayed by 2 to 4 weeks compared to the actual calendar date) with eggs hatching slightly later. Although these effects were in the expected direction, their small effect size of only 1.4–2.5 days delay combined with the lack of an (ordered) effect for the other photoperiod treatments likely indicates that photoperiod received at the egg stage plays a very minor role in regulating the seasonal timing of egg hatching in the winter moth. Indeed, in both experiments, the observed minor photoperiod effects could alternatively be explained by small unintended differences in experimental temperature between treatment replicates rather than photoperiod. As results are similar between the two experiments, we are confident that they are comparable, despite being performed 13 years apart with different equipment to manipulate the photoperiod and temperature that the eggs received.

Even though winter moth eggs are laid on bare tree branches (Varley et al. 1973) and are thus exposed to photoperiod during their entire development, it could be that developing embryos only start using photoperiodic information close to hatching. During most of their development, winter moth eggs are an opaque orange and embryos are nestled deep inside the egg (Gaumont 1950; van Dis et al. 2021). Eggs become transparent only very close to hatching, which is when they might start responding to photoperiod. Indeed, winter moth egg hatching has previously been observed to follow a circadian rhythm (Embree 1970) similar to other insects (Saunders 2002) and responding to photoperiod only at the end of egg development might also explain the small 1.4–2.5 days delay we observed for some of the delayed photoperiod treatments.

Compared to the overriding eight-fold larger temperature effect on egg development time (for arguably a smaller treatment difference of on average 1.36 °C between warm and cold treatments compared to a day length difference of 2 h in mid-March between photoperiod treatments), we would argue that photoperiod received at the egg stage does not play a substantial role in regulating the seasonal timing of egg hatching. In fact, the small photoperiod effects we found were outweighed by both between- and within-clutch variation in egg development time (Fig.S3). Nevertheless, photoperiod received at a different life stage might still play a role, as previous work on the winter moth has found indications of maternal effects of photoperiod (Salis et al. 2017). The first stage of insect embryogenesis critically depends on the maternally set-up environment in the egg (Irvine 2020) and dormancy responses are often maternally regulated in insects (Mousseau and Dingle 1991). For example, egg diapause is maternally induced in the silk moth, *B. mori* (Kogure 1933). In the winter moth, previous work indicated that photoperiod received by the mother can carry over into the next generation: mothers that received an early season photoperiod as caterpillars laid eggs that took less time to develop and vice versa, with an effect size of up to 10 days depending on the temperature treatment the eggs received (Salis et al. 2017). This trans-generational photoperiod response might be linked to the nutritional status of the mother (i.e. whether she was timed well to budburst as a caterpillar (van Asch et al. 2010), but the causal mechanism behind this maternal effect remains unclear.

Because climate change affects ambient temperature but not photoperiod, the relative importance of temperature and photoperiod as cues has important implications for climate change adaptation. Importantly, phenological traits mostly regulated by temperature are expected to immediately shift under climate change and such temperature-only controls of phenology might be common in moth species (e.g. in at least 34% of 112 analysed Finnish species, (Valtonen et al. 2011). While development rate is temperature dependent in all insect species (Nedved 2009), it might be that insects with obligate diapause (i.e. where diapause does not need to be induced by environmental cues) are more likely to have temperature-only controls of phenology, while species with facultative diapause often also rely on photoperiod as a cue to regulate dormancy (Denlinger 2002). Indeed, photoperiod regulation of diapause induction, maintenance, and termination has mostly been reported for facultative diapausers (e.g. Brunnarius and Dumortier 1984; Wang et al. 2009; Yang et al. 2014), while studies in obligate diapausers tend to focus on the effect of temperature only (e.g. Doherty et al. 2018; Gray et al. 2001). But to properly test this pattern, more experiments investigating the effect of photoperiod on the phenology of obligate diapausers are needed. In addition, it is important to identify where in the life cycle climate change-induced selection acts in order to understand which environmental cues are important for adaptation. This importance is illustrated by the few examples we have of insects evolving under climate change (Merilä and Hendry 2014): so far, genetic changes to the photoperiodic response were involved in pre-dormancy adaptations – e.g. the pitcher plant mosquito, *Wyeomyia smithii* (Bradshaw and Holzapfel 2001), and speckled wood butterfly, *Pararge aegeria* (Nielsen et al. 2023), where the photoperiodic response genetically changed to take advantage of the longer growing season. In contrast, genetic adaptation in the winter moth changed post-dormancy seasonal timing, involving changes to the temperature response (van Asch et al. 2007, 2013).

## Conclusion

Seasonal timing shifts are one of the most ubiquitous responses to climate change across taxa (Parmesan and Yohe 2003; Root et al. 2003; Thackeray et al. 2010). Elucidating which environmental cues regulate these timing responses is a crucial step in determining how populations can adapt to climate change. We conclude that temperature has an overriding role compared to photoperiod in regulating the seasonal timing of winter moth egg hatching. These relative contributions of temperature and photoperiod could have important implications for climate change adaptation. So far, we know of only few species that have evolved under climate change (Catullo et al. 2019; Merilä and Hendry 2014), but selection often seems to target seasonal timing responses (Bradshaw and Holzapfel 2008; Visser and Gienapp 2019). Future work should take care in determining both the specific selection pressure that climate change exerts on the seasonal timing trait as well as its underlying mechanism, as we might expect different targets of selection depending on the relative contribution of different environmental cues.

### Supplementary Information

Below is the link to the electronic supplementary material.

Supplementary file1 (DOCX 731 KB)

## Data Availability

All data and scripts used for analysis are available in the Dryad digital repository https://doi.org/10.5061/dryad.2v6wwpzvp (van Dis et al. 2024). Analysis scripts can also be found on GitHub: https://github.com/NEvanDis/WM_photoperiod.
