# Supplementary material for: Temperature has an overriding role compared to photoperiod in regulating the seasonal timing of winter moth egg hatching
Source: Oecologia. 2024 Mar 23;204(4):743–50. doi: 10.1007/s00442-024-05535-w (PMC11062991; doi:10.1007/s00442-024-05535-w)
Supplement: Supplementary file 1 — Supplementary file1 (DOCX 731 KB) [file 442_2024_5535_MOESM1_ESM.docx]

# Temperature has an overriding role compared to photoperiod in regulating the seasonal timing of winter moth egg hatching

Natalie E. van Dis^1,2,3^ǂ, Lucia Salis^1,2^ & Marcel E. Visser^1,2^

ǂ **Corresponding author:** Natalie E. van Dis, n.vandis@nioo.knaw.nl

ORCID ID: 0000-0002-9934-6751

**Journal name:** Oecologia

**Article DOI:** https://doi.org/10.1007/s00442-024-05535-w

**Keywords:** climate change, phenological mismatch, insect dormancy, diapause, *Operophtera brumata*

# **Supplements**


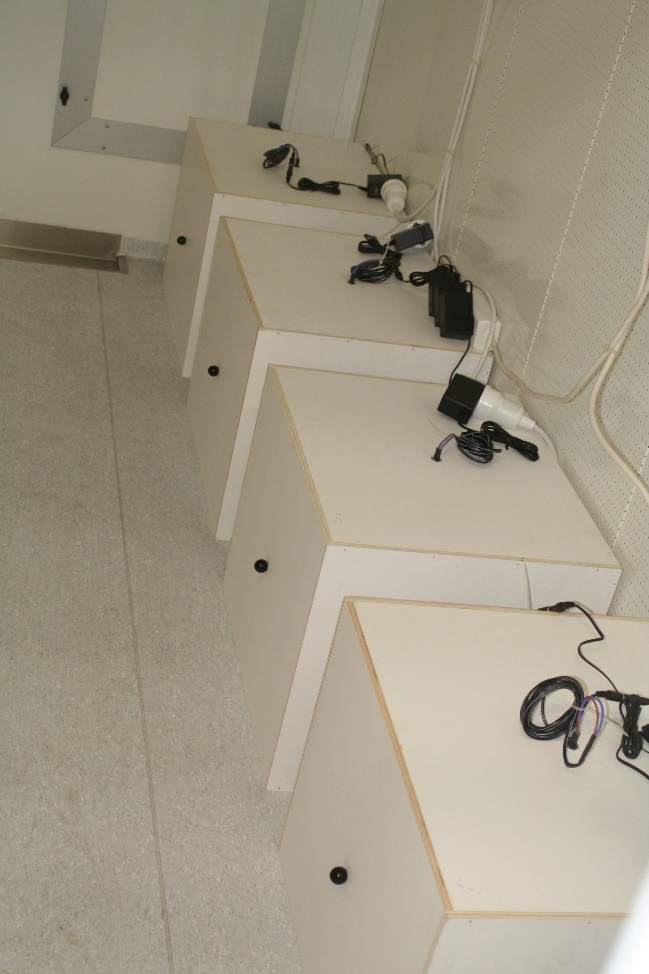

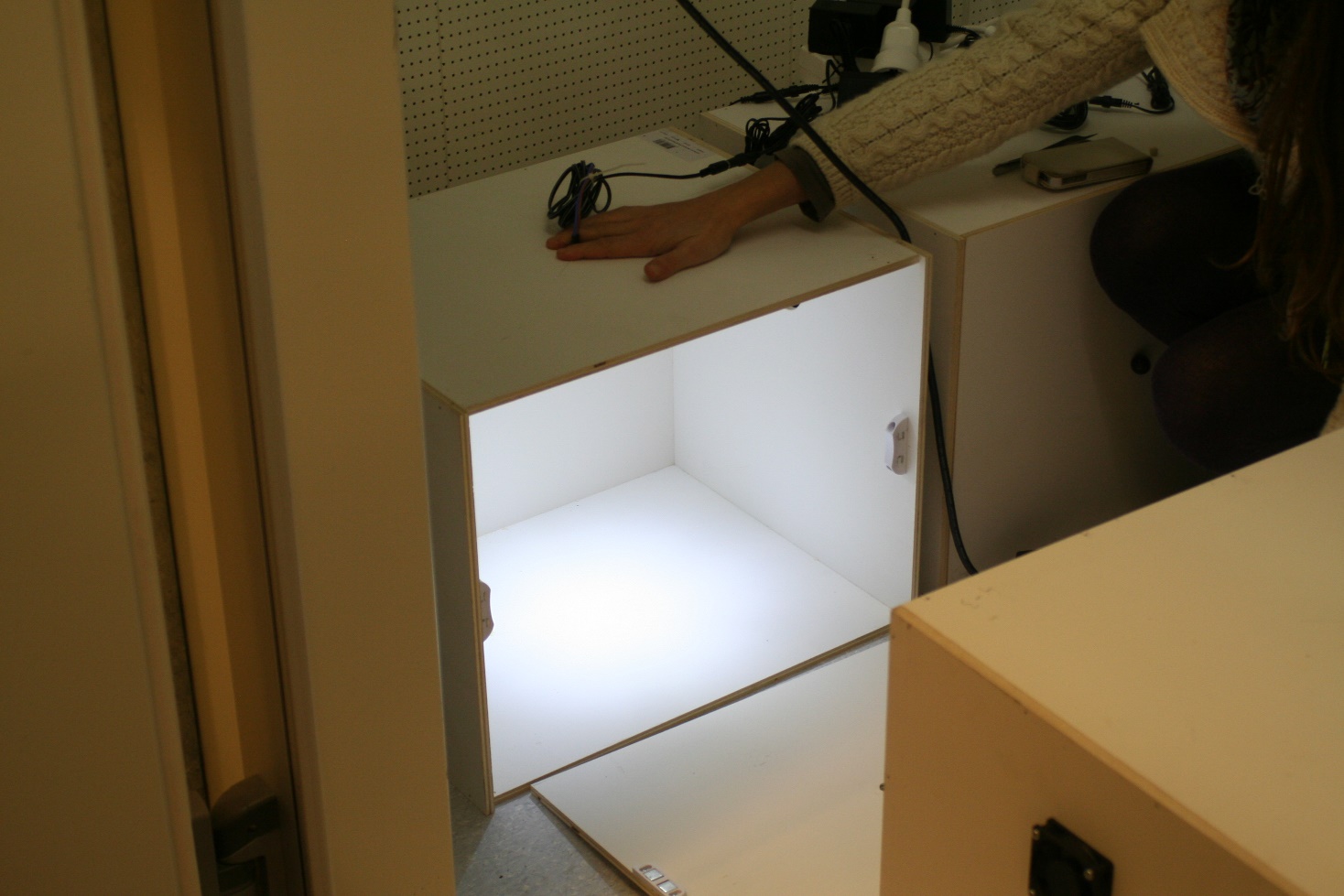


**Figure S1 – Experimental set-up for the photoperiod experiment.** Sub-clutches were kept in the same climate room at a constant 10°C, but each replicate per treatment was housed in an individual, ventilated box (i.e. 15 boxes, 30x30x30cm, ventilator on the left side of the box not visible in the picture) equipped with a light bulb (Goobay LED 2W/6200K, white, 1095-1132 lux as measured at the bottom of the boxes). Lamps were turned on and off automatically via an electronic timer.


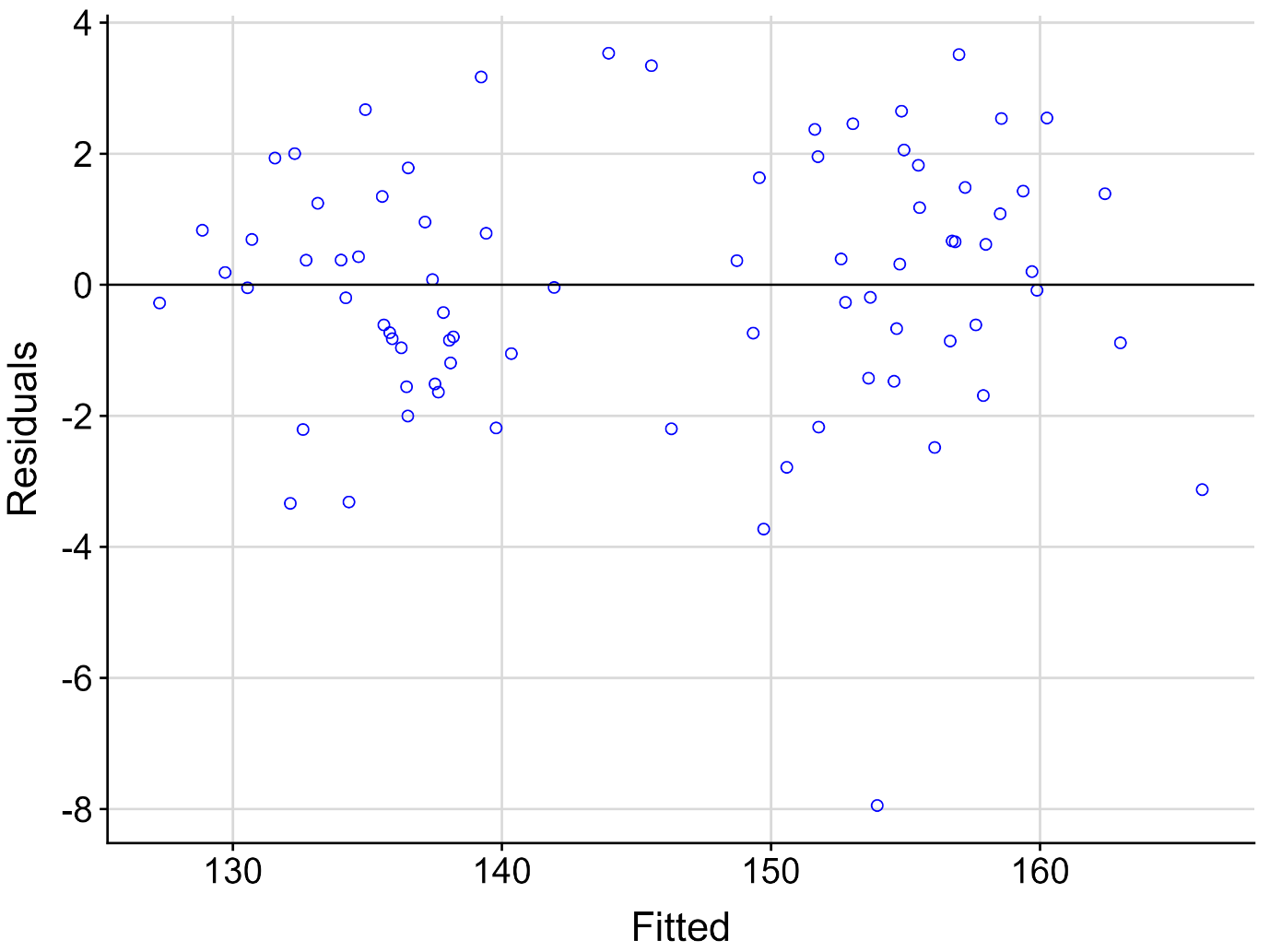


**Figure S2 – Residuals vs. fitted plot of the photoperiod-temperature experiment analysis.** We observed one major outlier in the residuals of the linear mixed model testing the effect of photoperiod and temperature and their interaction on egg development time. We excluded this outlier because its model residual was more than 3 standard deviations (SD) above the mean. This outlier resulted from one sub-clutch having a very different egg development time compared to the other sub-clutches of the same female (Female 10 in Figure S4).

**
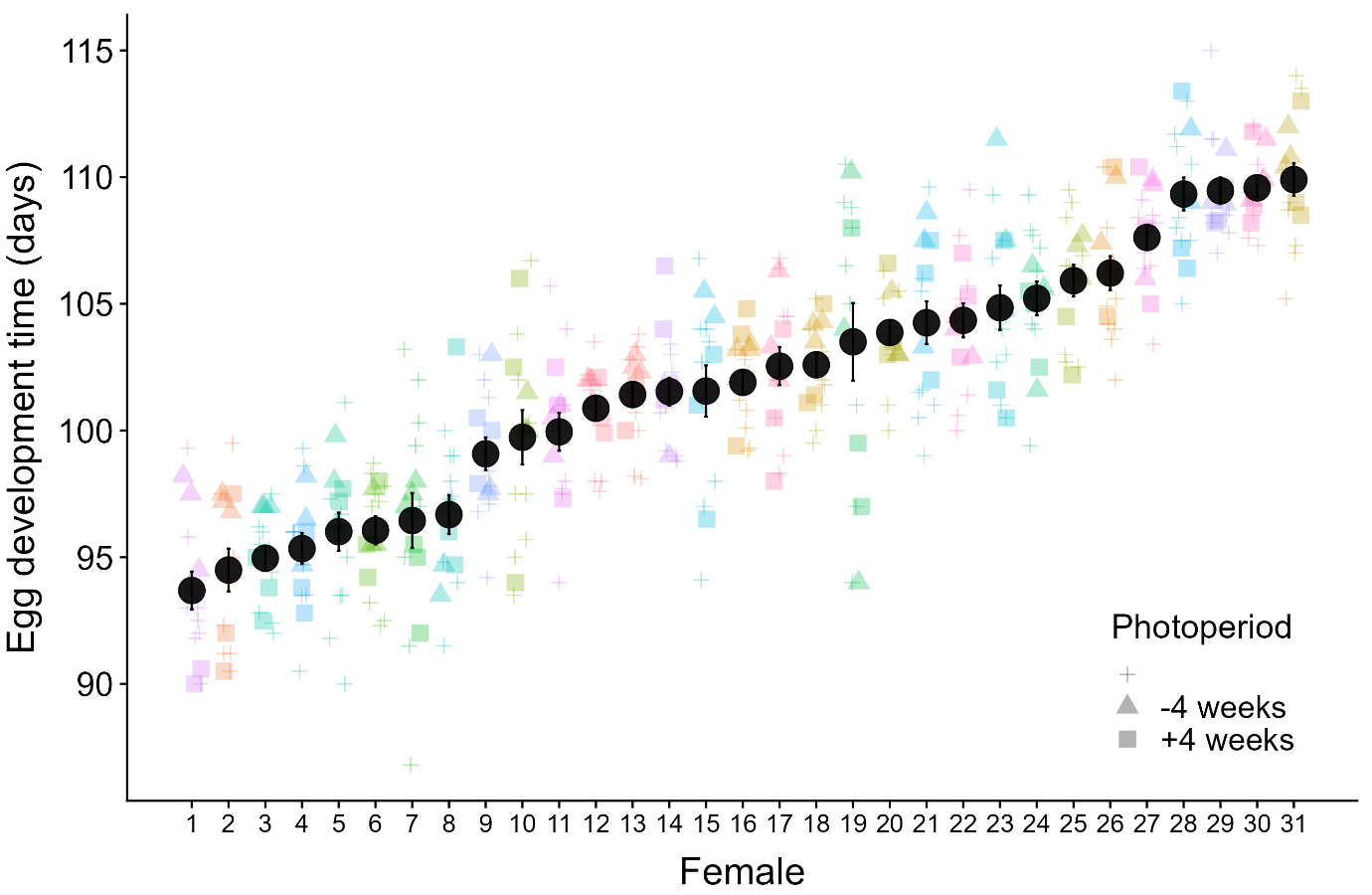
Figure S3 – Between-clutch variation in egg development time in the photoperiod experiment.** Mean egg development times ±SE are plotted for each female. Measures for individual sub-clutches are plotted in the background, coloured by female and with the shape indicating sub-clutches from the two most extreme photoperiod treatments (triangles=[-4 weeks]; squares=[+4 weeks], plus sign=other treatments). We observed large variation in egg development time between clutches laid by different females, ranging from 94 to 110 days (16 days max. difference), but there is no clear photoperiod effect visible per clutch.


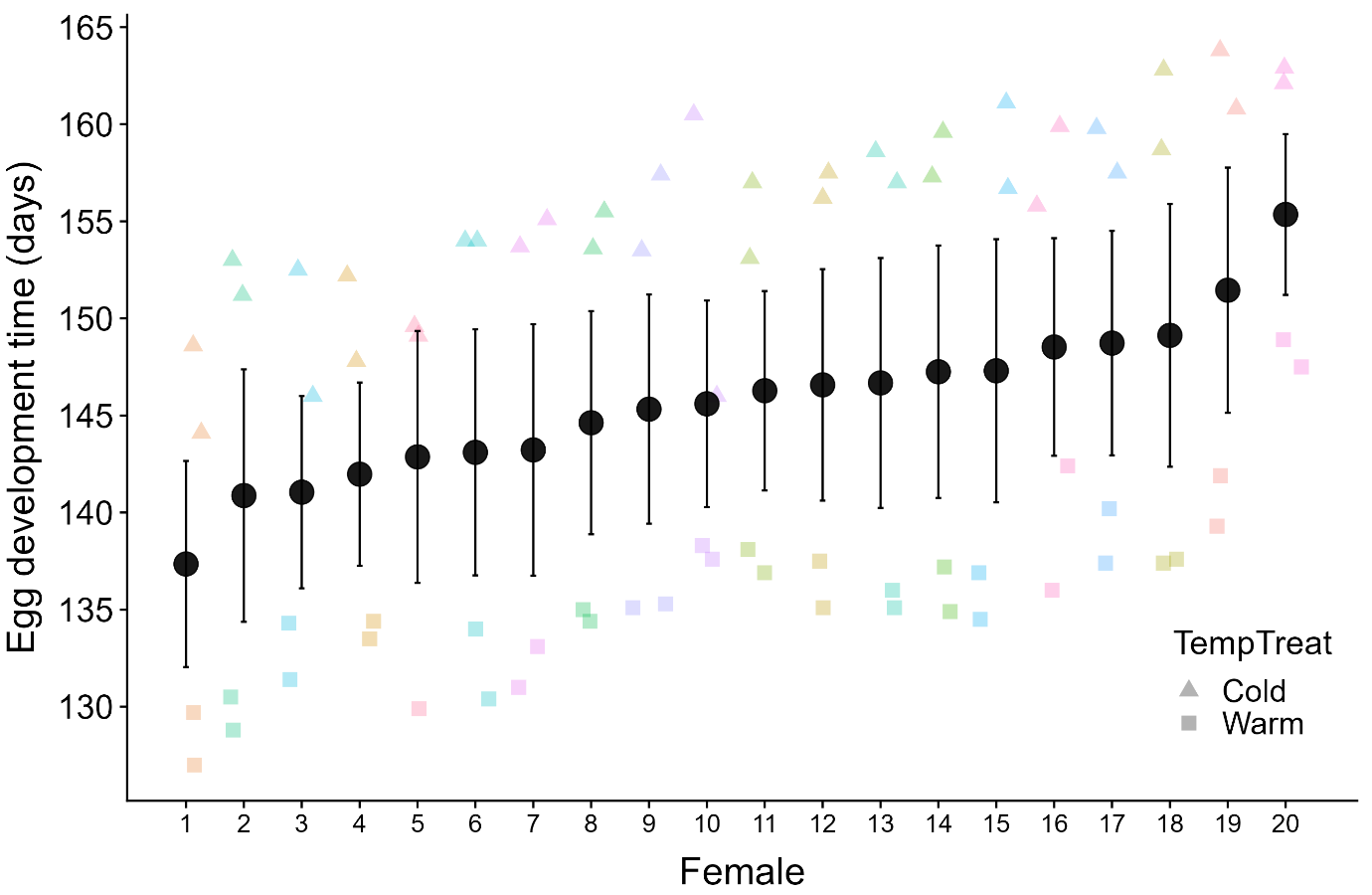


**Figure S4 – Between-clutch variation in egg development time in the photoperiod-temperature experiment.** Mean egg development times ±SE are plotted for each female, with individual sub-clutches plotted in the background, coloured by female and with the shape indicating temperature treatment per sub-clutch (Cold or Warm). We observed large variation in egg development time between clutches laid by different females, ranging from 137 to 155 days (18 days max. difference). But, for each clutch, sub-clutches were clearly differentiated between the cold and the warm treatment, hatching earlier in the warm and later in the cold treatment.

**Table S1 – ANOVA results for linear mixed effect models.** Results for each fixed effect are shown for the photoperiod experiment and the photoperiod-temperature experiment, reporting the sum of squares (SumSq) and mean squares (MeanSq), degrees of freedom (NumDF and DenDF), F-value, and P-value (P<0.05 in bold; method: Satterthwaite's type III ANOVA with lmerTest:anova()). Each model also included FemaleID as random intercept.

| **Model parameter** | **SumSq/MeanSq** | **NumDF** | **DenDF** | **F-value** | **P-value** |
| --- | --- | --- | --- | --- | --- |
| *Photoperiod experiment* | |  |  |  |  |
| Photoperiod treatment | 152.79/38.20 | 4 | 427.01 | 4.95 | **<0.001** |
| *Photoperiod-temperature experiment* | |  |  |  |  |
| Temperature treatment | 7750.68/7750.68 | 1 | 55.05 | 2123.86 | **<0.001** |
| Photoperiod treatment | 4.08/4.08 | 1 | 55.04 | 1.12 | 0.29 |
| Temp:Phot treatment | 79.76/79.76 | 1 | 55.05 | 21.86 | **<0.001** |

**Table S2 – Post-hoc results for photoperiod experiment.** We compared mean egg development time between photoperiod treatments, reporting the results for each contrast including the estimate (in days), standard error (Std.Err), degrees of freedom (DF), t.ratio, and P-value (P<0.05 in bold, adjusted for multiple-testing with the Tukey method).

| **Contrast** | **Estimate** | **Std.Err.** | **DF** | **t.ratio** | **P-value** |
| --- | --- | --- | --- | --- | --- |
| [-4 weeks] - [ -2 weeks] | 1.47 | 0.41 | 427 | 3.60 | **0.003** |
| [-4 weeks] - [ 0 weeks] | 1.48 | 0.41 | 427 | 3.61 | **0.003** |
| [-4 weeks] - [+2 weeks] | 0.60 | 0.41 | 427 | 1.46 | 0.59 |
| [-4 weeks] - [+4 weeks] | 1.23 | 0.41 | 427 | 3.01 | **0.02** |
| [-2 weeks] - [ 0 weeks] | 0.01 | 0.41 | 427 | 0.02 | 1.00 |
| [-2 weeks] - [+2 weeks] | -0.88 | 0.41 | 427 | -2.14 | 0.20 |
| [-2 weeks] - [+4 weeks] | -0.24 | 0.41 | 427 | -0.59 | 0.98 |
| [ 0 weeks] - [+2 weeks] | -0.88 | 0.41 | 427 | -2.15 | 0.20 |
| [ 0 weeks] - [+4 weeks] | -0.25 | 0.41 | 427 | -0.61 | 0.97 |
| [+2 weeks] - [+4 weeks] | 0.63 | 0.41 | 427 | 1.55 | 0.53 |

**Table S3 – Observed mean experimental temperatures.** We report the observed mean experimental temperatures and the standard deviation (Std.Dev), calculated for each experiment from the start of the experiment to the median hatch date per treatment combination. Experimental temperatures slightly deviated between replicate temperature treatments, both for the photoperiod experiment (all treatments constant 10°C ±0.1-0.3) and for the photoperiod-temperature experiment cold (±0.1°C) and warm treatments (±0.4°C).

| **Treatment** | **Mean temperature (°C)** | **Std.Dev.** |
| --- | --- | --- |
| *Photoperiod experiment* |  |  |
| [-4 weeks] | 10.55 | 0.40 |
| [-2 weeks] | 10.84 | 0.50 |
| [ 0 weeks] | 10.68 | 0.45 |
| [+2 weeks] | 10.64 | 0.41 |
| [+4 weeks] | 10.85 | 0.43 |
| *Photoperiod-temperature experiment* | |  |
| Cold - [-2 weeks] | 3.89 | 3.55 |
| Cold - [+2 weeks] | 3.98 | 3.58 |
|  |  |  |
| Warm - [-2 weeks] | 5.48 | 3.99 |
| Warm - [+2 weeks] | 5.10 | 4.00 |

**Table S4– Post-hoc results for photoperiod-temperature experiment.** We compared mean egg development time between photoperiod treatments within temperature treatment and vice versa, since the interaction between photoperiod and temperature treatments was significant (P<0.001, Table S1). Reported results include for each contrast the estimate (in days), standard error (Std.Err), degrees of freedom (DF), t.ratio, and P-value (P<0.05 in bold).

| **Contrast** | **Estimate** | **Std.Err.** | **DF** | **t.ratio** | **P-value** |
| --- | --- | --- | --- | --- | --- |
| **Cold:** [-2 weeks] - [+2 weeks] | 2.49 | 0.61 | 55.06 | 4.05 | **0.001** |
| **Warm:** [-2 weeks] - [+2 weeks] | -1.57 | 0.61 | 55.06 | -2.56 | 0.053 |
|  |  |  |  |  |  |
| **[-2 weeks]:** Cold - Warm | 22.06 | 0.60 | 55.00 | 36.52 | **<0.001** |
| **[+2 weeks]:** Cold - Warm | 18.00 | 0.63 | 55.13 | 28.79 | **<0.001** |
